# Validation of combined carcinoembryonic antigen and glucose testing in pancreatic cyst fluid to differentiate mucinous from non-mucinous cysts

**DOI:** 10.1007/s00464-022-09822-6

**Published:** 2023-01-19

**Authors:** Myrte Gorris, Frederike Dijk, Arantza Farina, Johannes B. Halfwerk, Gerrit K. Hooijer, Selma J. Lekkerkerker, Rogier P. Voermans, Mattheus C. Wielenga, Marc G. Besselink, Jeanin E. van Hooft

**Affiliations:** 1 Department of Gastroenterology and Hepatology, Amsterdam UMC, Location University of Amsterdam, Amsterdam, The Netherlands; 2 Amsterdam Gastroenterology Endocrinology Metabolism, Amsterdam, The Netherlands; 3 Department of Surgery, Amsterdam UMC, Location University of Amsterdam, Amsterdam, The Netherlands; 4 Cancer Center Amsterdam, Amsterdam, The Netherlands; 5 Department of Pathology, Amsterdam UMC, Location University of Amsterdam, Meibergdreef 9, 1105 AZ Amsterdam, The Netherlands; 6 Department of Gastroenterology and Hepatology, Leiden University Medical Center, Leiden, The Netherlands

**Keywords:** Pancreatic cystic neoplasms, Pancreatic cyst fluid, Carcinoembryonic antigen (CEA), Glucose, Diagnostic accuracy, Hepatobiliary tract and spleen, Endoscopy: Endoscopic ultrasound diagnostic and therapeutic

## Abstract

**Background:**

More accurate diagnosis of mucinous cysts will reduce the risk of unnecessary pancreatic surgery. Carcinoembryonic antigen (CEA) and glucose in pancreatic cyst fluid (PCF) can differentiate mucinous from non-mucinous pancreatic cystic neoplasms (PCN). The current study assessed the value of combined CEA and glucose testing in PCF.

**Methods:**

Cross-sectional validation study including prospectively collected PCF from patients undergoing endoscopic ultrasonography-guided fine-needle aspiration (EUS-FNA) and pancreatic surgery. We performed laboratory measurements for CEA and glucose and measured glucose levels by a hand glucometer. Primary outcome was diagnostic accuracy evaluated by receiver operator curves (ROC), sensitivity, specificity, positive, and negative predictive value (PPV, NPV).

**Results:**

Overall, PCF was collected from 63 patients, including 33 (52%) with mucinous and 30 (48%) with non-mucinous PCN. Histopathology (*n* = 36; 57%), cytopathology (*n* = 2; 3%), or clinical and/or radiological diagnosis (*n* = 25; 40%) was used as reference standard. Combined CEA (cut-off ≥ 192 ng/ml) and laboratory glucose testing (cut-off ≤ 50 mg/dL) reached 92% specificity and 48% sensitivity, whereas either positive CEA (cut-off ≥ 20 ng/ml) or glucose testing (cut-off ≤ 50 mg/dL) showed 97% sensitivity and 50% specificity. Sensitivity and specificity were 80% and 68% for CEA ≥ 20 ng/mL versus 50% and 93% for CEA ≥ 192 ng/mL (the conventional cut-off level). Laboratory and glucometer glucose both reached 100% sensitivity and 60% and 45% specificity, respectively. None of the biomarkers and cut-offs reached a PPV exceeding 90%, whereas both glucose measurements had a NPV of 100% (i.e., high glucose excludes a mucinous cyst).

**Conclusion:**

Combined CEA and glucose testing in PCF reached high specificity and sensitivity for differentiating mucinous from non-mucinous PCN. Glucose testing, whether alone or combined with the new CEA cut-off (≥ 20 ng/mL), reached > 95% sensitivity for mucinous cysts, whereas only glucose reached a NPV > 95%.

**Graphical abstract:**

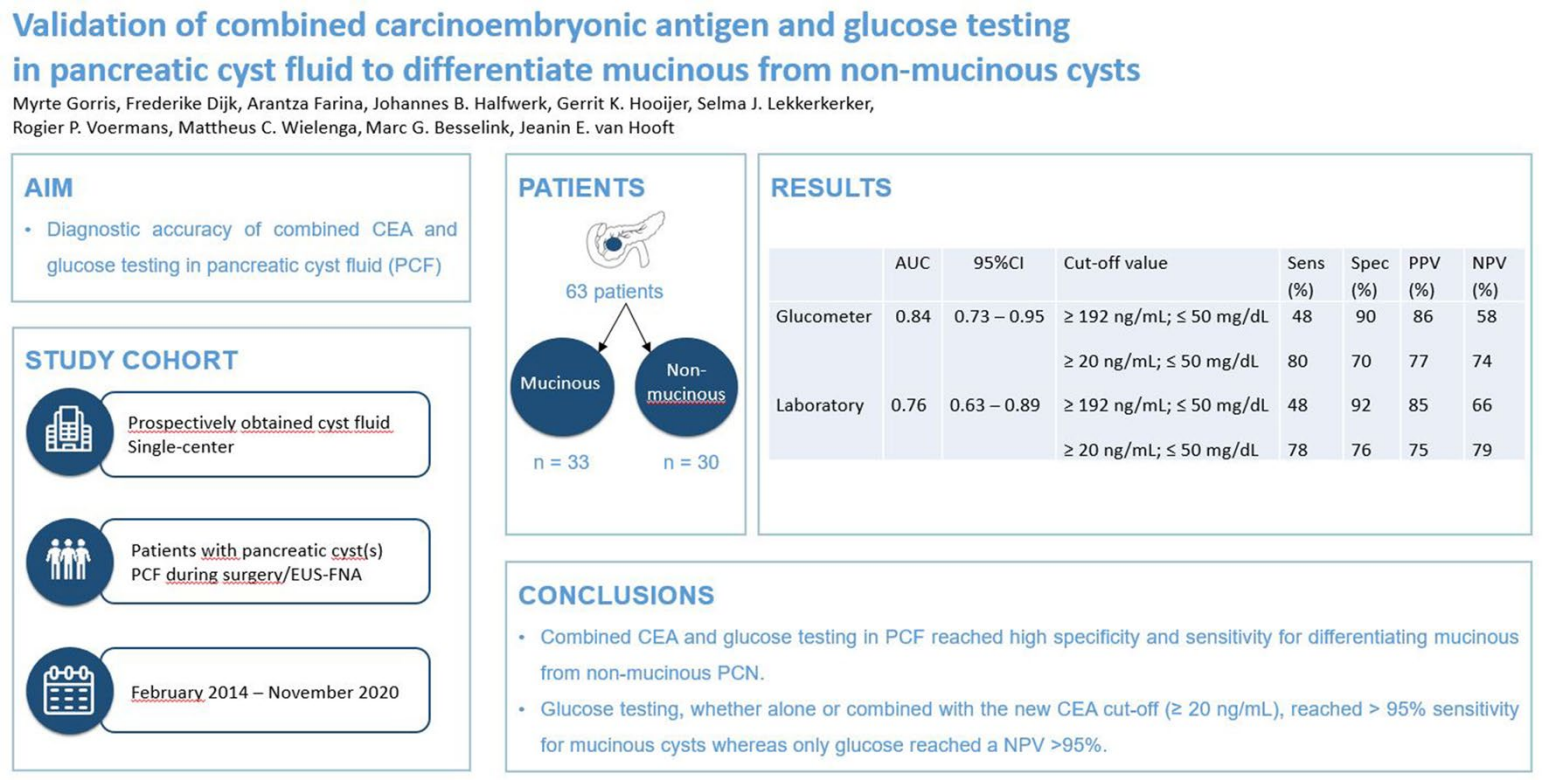

**Supplementary Information:**

The online version contains supplementary material available at 10.1007/s00464-022-09822-6.

Pancreatic cystic neoplasms (PCN) are increasingly detected incidentally on radiological imaging, with a reported weighted incidence of up to 49% in the general population, increasing with age [1]. PCN compromise a heterogeneous group of lesions, ranging from benign to (pre)malignant entities [2]. Mucinous PCN are considered (pre)malignant and thus require follow-up or surgical resection according to current international guidelines [3, 4]. On the other hand, non-mucinous PCN do not require surveillance or intervention. Thus, in order to prevent unnecessary surgery with associated mortality, morbidity, and costs, it is essential to accurately distinguish mucinous from non-mucinous PCN. However, differentiating different types of PCN remains challenging in daily clinical patient care. Even if best practice according to the clinical guidelines is applied, only 72% of PCN are diagnosed correctly and adequate differentiation between mucinous and non-mucinous PCN is made in 86% [5]. Thus, improving accurate distinction between mucinous and non-mucinous PCN is essential in order to: (1) prevent lifelong futile follow-up of non-mucinous cysts, (2) allow for timely intervention in (pre)malignant PCN, and (3) avoid futile major abdominal surgery in wrongly diagnosed mucinous PCN.

Biochemical testing of pancreatic cyst fluid (PCF) obtained by endoscopic ultrasound with fine-needle aspiration (EUS-FNA) is often used during diagnostic workup as it improves diagnostic accuracy in differentiating mucinous from non-mucinous PCN [3]. From a clinical perspective, easily accessible biochemical markers to accurately analyze PCF are a crucial necessity in daily patient care. Carcinoembryonic antigen (CEA) is frequently used for this purpose and has the ability to distinguish mucinous from non-mucinous PCN with a sensitivity of 52–73% and a specificity of 77–89% using a cut-off value of 192 ng/mL [6–8]. Nevertheless, the optimal cut-off value of CEA is still under debate, as underlined by data from a recently performed individual patient meta-analysis in 365 patients showing that a lower cut-off value of 20 ng/mL achieved the highest diagnostic accuracy [sensitivity 91% (95%CI 88–94%); specificity 85% (95%CI 72–93%)] [9].

Another biochemical PCN-biomarker is glucose, a relatively novel and promising biomarker due to its widespread availability. A recently published meta-analysis reported a diagnostic accuracy of 94% for differentiating mucinous from non-mucinous PCN, although the included studies used heterogeneous forms of measurements (e.g., laboratory measurements, glucometer testing) and its use is not yet standardized in clinical practice [10]. In this validation study, we aimed to assess the diagnostic accuracy of combined CEA and glucose testing to discriminate mucinous from non-mucinous PCN.

## Materials and methods

### Study design and participants

We performed a cross-sectional validation study on prospectively collected PCF samples to investigate the diagnostic accuracy of CEA and glucose in differentiating mucinous from non-mucinous PCN. This study was performed in accordance with the Standards for Reporting Diagnostic accuracy studies (STARD) guidelines for diagnostic accuracy studies [11]. The study was approved by the institutional review board of the Amsterdam UMC. All patients provided informed consent prior to the procedure for storage of residual material. Consecutive patients ≥ 18 years who underwent EUS-FNA or pancreatic surgery for a pancreatic cystic lesion between February 2014 and November 2020 and in whom PCF was obtained were eligible for inclusion. Patients were excluded if no PCF was available, if patients were diagnosed with extra pancreatic disease (e.g., ampullary adenoma), or in case PCN-derived pancreatic cancer could not be excluded.

### Data collection

PCF samples were prospectively collected during either EUS-FNA or at the pathology grossing-room after pancreatic surgery. EUS procedures were performed by or under direct supervision of a specialized endosonographist with the use of standard techniques. PCF was obtained at the discretion of the endosonographist by aspirating cystic fluid directly from the lesion. In patients who underwent pancreatic surgery, PCF was aspirated directly from the cystic lesion during processing of the resection specimen at the pathology ward. Samples were transferred to the pathology department and stored at a temperature of − 80° C. In case multiple samples were obtained from the same patient, the sample obtained during EUS was used for the analysis. The surgical cohort consisted merely of patients with histopathological confirmation of diagnosis. Clinical data were retrospectively collected from the electronic patient files.

### Tumor markers

CEA was either prospectively determined as part of clinical care, or an aliquot of the PCF sample was thawed at 37° C and transferred to the clinical laboratory. All measurements were performed by the clinical laboratory of the Amsterdam UMC. Measurements took place on the same day (electrochemiluminescence using enzyme-labeled sandwich immunoassay, Cobas e602, Roche Diagnostics). Also for glucose measurements frozen samples were quickly thawed at 37° C. The StatStrip® glucometer (Nova Biomedical Massachusetts, USA) was used to determine glucometer glucose levels in an aliquot of our PCF samples. The StatStrip® displayed all samples with a glucose level < 0.6 mg/L (= 10.8 mg/dL) as ‘low’. For numerical comparisons, we assigned/denoted these samples with a glucose level of 0.5 mg/L (= 9 mg/dL). Afterward, samples were transferred to the clinical laboratory, where the measurements took place on the same day (spectophotometric assessment using Hexokinase, Cobas c702, Roche Diagnostics). All researchers performing the measurements were blinded for the final diagnoses.

### Outcomes

Primary outcome was to evaluate the diagnostic accuracy of combined CEA and glucose testing in PCF to distinguish mucinous from non-mucinous PCN, evaluated by using receiver operating curves (ROC). ROC were obtained for two cut-off values of CEA (≥ 192 ng/mL and ≥ 20 ng/mL) and for two glucose measuring tools (i.e., laboratory and glucometer measurements). As secondary outcomes, sensitivity, specificity, positive predictive value (PPV), and negative predictive value (NPV) were evaluated. To evaluate the accuracy of combined testing, tests were categorized as positive if: (a) both CEA and glucose were positive (‘CEA and glucose positive’), or (b) if either CEA or glucose were positive (‘CEA or glucose positive’). Histopathological proof, cytopathological proof, or clinical and/or radiological follow-up were used as reference standard. If clinical and/or radiological follow-up was used as reference standard, all available diagnostics (e.g., clinical characteristics, radiological imaging, follow-up information) were discussed in a multidisciplinary pancreatic cyst meeting to determine the most likely clinical diagnosis. Mucinous PCN consisted of intraductal papillary mucinous neoplasm (IPMN) and mucinous cystadenoma (MCN). Pseudocysts, pancreatic neuroendocrine tumor, serous cystadenoma (SCN), ciliated foregut cyst, and lymphatic malformation were categorized as non-mucinous PCN.

### Statistical analysis

Continuous data were reported as median and interquartile range (IQR) and the Mann–Whitney *U* test was used to compare continuous data between the groups. Categorical data were reported as frequency or percentage. Chi-square test (or Fisher’s exact test where appropriate) was used to compare categorical data. Fisher’s exact test was used to compare areas under the curve (AUC). *p* values of < 0.05 were considered to be statistically significant.

## Results

### Baseline and disease characteristics

**Fig. 1:**
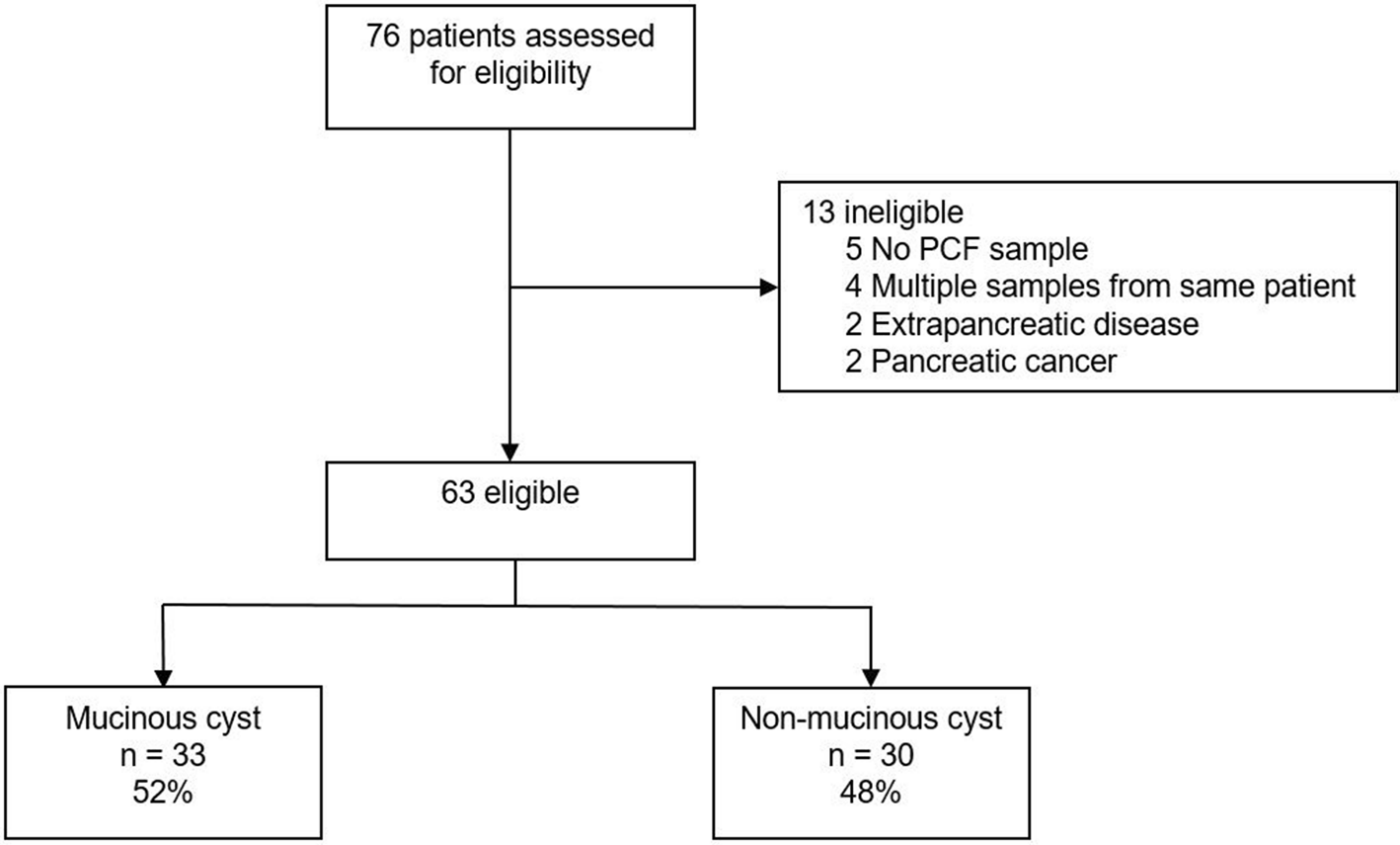
Study inclusion. *n* number, *PCF* pancreatic cyst fluid

In total, PCF was obtained from 76 patients, of which 63 patients were included in the current analysis. Thirty-six patients (57%) underwent surgical resection (Fig. 1). The study cohort consisted of 31 (49%) females and had a median age of 65 (IQR 51–71) years. Thirty-three (52%) patients had a mucinous cyst and 30 (48%) had a non-mucinous cyst. CEA was prospectively determined as part of clinical care in 24/63 patients (38%). Diagnoses were confirmed by histopathology (*n* = 36; 57%), cytopathology (*n* = 2; 3%), or clinical and/or radiological findings (*n* = 25; 40%). IPMN was the most frequent diagnosis (*n* = 23; 70%) in patients with mucinous PCN, whereas most non-mucinous PCN were SCN (*n* = 13; 43%). The combination of PCF-CEA, PCF-glucometer glucose, and PCF-laboratory glucose levels was available in the majority of patients (*n* = 46; 73%). In the other patients, either a combination of two biochemical markers (*i* = 12; 19%) or only CEA was available (*n* = 5; 8%). An overview of baseline and disease characteristics is displayed in Table 1.

**Table 1:** Baseline and disease characteristics

	Mucinous *n* = 33	Non-mucinous *n* = 30	*p* value
A. Baseline characteristics			
Female, *n* (%)	20 (61)	11 (37)	0.06
Age in years, median (IQR)	67 (54–72)	60 (43–71)	0.34
Serum CA 19.9 in U/mL, median (IQR)^a^	19 (11–33)	10 (9–14)	0.11
Cyst location, *n* (%)			0.47^b^
Head	17 (51)	15 (50)	
Body	4 (12)	7 (23)	
Tail	12 (36)	6 (20)	
Multifocal	–	2 (7)	
Biochemical marker availability, *n* (%)			0.30^b^
CEA, glucometer, and laboratory glucose	24 (73)	22 (73)	
CEA and glucometer	4 (12)	3 (10)	
CEA and laboratory glucose	1 (3)	4 (13)	
CEA only	4 (12)	1 (3)	
B. Disease characteristics			
Final diagnosis, *n* (%)			< 0.001^b^
IPMN	23 (70)	–	
MCN	10 (30)	–	
SCN	–	13 (43)	
Pseudocyst	–	12 (40)	
pNET	–	2 (7)	
SPN	–	1 (3)	
Other, benign^c^	–	2 (7)	
Confirmation of diagnosis, *n* (%)			< 0.001**b**
Clinical follow-up	6 (18)	19 (63)	
Cytopathology	1 (3)	1 (3)	
Histopathology	26 (79)	10 (33)	

Percentages might not sum to 100% because of rounding

*CA* cancer antigen, *CEA* carcinoembryonic antigen. *IPMN* intraductal papillary mucinous neoplasm, *IQR* interquartile range, *MCN* mucinous cystic neoplasm, *n* number, *pNET* pancreatic neuroendocrine tumor, *SCN* serous cystadenoma, *SPN* solid papillary neoplasm

^a^CA 19.9 was missing in 44 patients (70%)

^b^Fisher’s exact test was used

^c^One patient had a ciliated foregut cyst and one patient was diagnosed with a lymphatic malformation

### Combined CEA and glucose testing

Combined testing of CEA and glucometer glucose reached an AUC of 0.84 (95%CI 0.73–0.95) for differentiating mucinous from non-mucinous PCN, compared to an AUC of 0.76 (95%CI 0.63–0.89) for combined CEA and laboratory glucose testing (Table 2)*.* The combination of either CEA (≥ 20 ng/mL) or glucose (≤ 50 mg/dL) reached highest sensitivity (97% for glucometer and 96% for laboratory glucose, Table 2). Combined testing with both CEA (≥ 192 ng/mL) and glucose (≤ 50 mg/dL) reached highest specificity (90% for glucometer and 92% for laboratory glucose). When focusing on the surgical cohort, the same trend was observed (*Supplementary Table S1)*. All possible combinations of CEA and glucose testing were analyzed and results are provided in Table 2 (clinical cohort) and *Table S1* (surgical cohort).

**Table 2:** Accuracy of CEA and glucose to differentiate mucinous from non-mucinous PCN

	AUC	95%CI	*p* value	Cut-off value	Sensitivity (%)	Specificity (%)	PPV (%)	NPV (%)
*CEA*	0.82	0.72–0.92	< 0.001	≥ 192 ng/mL	50	93	88	63
				≥ 20 ng/mL	80	68	73	76
*Glucose*								
Glucometer	0.76	0.62–0.90	0.001	≤ 50 mg/dL	100	45	70	100
Laboratory	0.73	0.59–0.87	0.004	≤ 50 mg/dL	100	60	70	100
*CEA or glucose positive*								
CEA or glucometer	N/A	N/A	N/A	≥ 192 ng/mL or ≤ 50 mg/dL ≥ 20 ng/mL or ≤ 50 mg/dL	93 97	61 50	72 68	89 93
CEA or laboratory glucose	N/A	N/A	N/A	≥ 192 ng/mL or ≤ 50 mg/dL ≥ 20 ng/mL or ≤ 50 mg/dL	90 96	64 50	73 67	86 93
*CEA and glucose positive*								
Glucometer	0.84	0.73–0.95	< 0.001	≥ 192 ng/mL and ≤ 50 mg/dL ≥ 20 ng/mL and ≤ 50 mg/dL	48 80	90 70	86 77	58 74
Laboratory	0.76	0.63–0.89	0.001	≥ 192 ng/mL and ≤ 50 mg/dL ≥ 20 ng/mL and ≤ 50 mg/dL	48 78	92 76	85 75	66 79

*AUC* Area under the curve, *CEA* carcinoembryonic antigen, *CI* confidence interval, *dL* demi-liter, *mg* milligram, *mL* milliliter, *N/A* not applicable, *Ng* nanogram, *NPV* negative predictive value, *PCN* pancreatic cystic neoplasm, *PPV* positive predictive value

### CEA cut-off and glucose measurement techniques

Glucometer testing failed in a total of 7/56 (13%) cases (2 mucinous; 5 non-mucinous), whereas laboratory glucose testing failed in 3/51 patients (6%; 2 mucinous and 1 non-mucinous), and CEA measurements failed in 5/63 (8%) of cases (3 mucinous; 2 non-mucinous). Median CEA was higher in mucinous [173 mmol/L (IQR 28–1165)] when compared to non-mucinous PCN [4 mmol/L (IQR 0.4–31), *p* < 0.001]. Median glucose levels of both glucometer [9.0 mg/dL (IQR 9.0–9.0) vs. 33.3 mg/dL (IQR 9.0–81.9), *p* < 0.001] and laboratory measurements [3.6 mg/dL (IQR 1.8–12.6) vs. 63.0 mg/dL (IQR 1.8–103.5), *p* = 0.001] were lower in mucinous than in non-mucinous PCN.

CEA reached an AUC of 0.82 (95%CI 0.72–0.92, Table 2) for differentiating mucinous from non-mucinous PCN. For a cut-off value of ≥ 192 ng/mL, a sensitivity of 50% and specificity of 93% was reached. Lowering the cut-off value to ≥ 20 ng/mL increased sensitivity to 80% while specificity decreased to 68% (Table 2). The same trend was observed in the surgical cohort (*n* = 40, *Supplementary table S1*). Glucometer glucose and laboratory glucose reached an AUC of 0.76 (95%CI 0.62–0.90) and 0.73 (95%CI 0.59–0.87), respectively (Table 2*).* Sensitivity and NPV was 100% for both glucose measuring techniques, whereas laboratory glucose reached higher specificity (60%) when compared to glucometer measurements (45%). In the surgical cohort, specificity and PPV for glucometer testing increased to 80% and 95%, respectively, compared to 83% and 95% for laboratory glucose. An overview of the test characteristics of the entire cohort is displayed in Table 2, whereas *Supplementary Table S1* showed the results in patients with a histological proven diagnosis.

## Discussion

This first validation study on the diagnostic accuracy for combined CEA and glucose testing in PCF to differentiate mucinous from non-mucinous PCN found that combined CEA and glucose testing reached high specificity and sensitivity. The new cut-off value of CEA (≥ 20 ng/mL) led to an increased sensitivity of 80%. Glucose testing showed 100% sensitivity and NPV, making it a well accessible biomarker which can be easily implemented in clinical practice.

Currently, two systematic reviews have reported on the diagnostic accuracy of glucose in PCF to distinguish mucinous from non-mucinous PCN [10, 12]. The first review by McCarty et al. also investigated the value of combined CEA and glucose testing, and reported no improvement in the diagnostic accuracy when compared to glucose alone (based on overlapping confidence intervals). However, only 4 studies were included for this analysis [10]. A more recent retrospective study in 102 patients, which was not included in the aforementioned meta-analysis, concluded that combined CEA and glucose testing reached an AUC of 0.94 (95%CI 0.88–0.99), with a sensitivity of 88% and a specificity of 93% [13]. Both systematic reviews reported a high sensitivity (91% and 90.5%) and specificity (86% and 88%) for glucose testing. Our results also showed high sensitivity of glucose (100% for both techniques), and in the subset of patients in our cohort with histopathological confirmed diagnosis, specificity was also comparable (83–86%). Nevertheless, as also stated by McCarty et al., glucose measurements are currently performed with different techniques [10]. Therefore, our study aimed to provide insight in the accuracy of both glucometer and laboratory glucose measurements. In line with a previous study by Zikos et al., our results showed that glucose can be accurately determined by both techniques [14]. Glucometer measurements are cheap and widely available, and may therefore serve as an easily accessible biochemical marker. Nevertheless, these results should be validated in larger cohorts.

The findings in the current study are in line with previous studies that compared the diagnostic accuracy of CEA using the conventional cut-off value of ≥ 192 ng/mL to other cut-off values to differentiate mucinous from non-mucinous PCN. These studies reported sensitivity rates ranging from 52% to 73% and specificity rates between 77 and 89% [6–8]. Nevertheless, the optimal cut-off value of CEA remains under debate, as underlined by a recently performed individual patient meta-analysis (full data not yet published). In this study by van Huijgevoort et al., a lower cut-off value of ≥ 20 ng/mL reached highest pooled sensitivity (91%) and specificity (85%) as compared to 67% and 76%, respectively, for a cut-off value of ≥ 192 ng/mL [9]. In the current study, we aimed to validate these findings. We observed an increased sensitivity yet decreased specificity when lowering the cut-off value of CEA. Thus, we were unable to validate the results reported in the abovementioned meta-analysis. This difference might be caused by the fact that we also included patients without histopathological confirmed diagnosis in our cohort. However, when analysis was restricted to only surgically treated patients (*n* = 40), specificity also decreased from 100 to 75%.

Another, more recent development in PCF analysis is the possibility to conduct next-generation sequencing for molecular analysis. A recently performed meta-analysis showed that the presence of mutations in KRAS and GNAS mutations have a high diagnostic accuracy (97%) for diagnosing mucinous PCN [15]. Although these results are promising, mutation sequencing is less widely accessible and experienced laboratory staff is a prerequisite to perform these techniques. Therefore, easily accessible biochemical markers remain a crucial necessity in clinical patient care.

This study has some limitations. First, the sample size was relatively small. Second, only a subset of our cohort had pathological confirmation of final diagnosis, thereby introducing the risk of confirmation bias. However, this cohort does reflect the patient population in common clinical practice. Furthermore, we created insight in the differences in diagnostic accuracy between the clinical and surgical cohort by providing separate analyses which showed no concerning differences. Third, the PCF samples were obtained in a single-center tertiary care setting, thereby impacting generalizability to other hospital settings. Fourth, PCF was obtained during EUS-FNA and surgical procedures, thereby possibly introducing heterogeneity in the samples, since surgical samples were transported to the grossing-room prior to collection of PCF and stored at − 80° C. Nevertheless, transportation time is short and it is therefore not likely that glucose and CEA levels changed significantly during this period.

Nevertheless, the strengths of this study consist of the use of a prospectively obtained cohort of PCF samples from a study population that represents daily clinical practice. In addition, endoscopic PCF samples were immediately frozen at − 80° C after collection, thereby minimizing the risk of degradation. Furthermore, we analyzed two glucose measurement techniques and showed results for different forms of combined CEA and glucose testing. As a consequence, this study provides insight in the diagnostic accuracy of different testing techniques and thus enables clinicians to deliberate on the most useful combination. The main advantage of combined CEA and glucose testing lies within the possibility to use specific combinations based on the most convenient result for an individual patient. For example, in a patient with multiple comorbidities and an indication for surgical resection, high specificity is especially important to confirm a mucinous cyst in which case combined testing with glucose and CEA (≥ 192 ng/mL) could be advocated. In contrast, in patients in whom follow-up might be stopped, there is need for a high sensitivity to rule out a mucinous cyst and a combined testing strategy with either a positive CEA (≥ 20 ng/mL) or a positive glucose (≤ 50 mg/dL) can be used. Larger cohort studies are however warranted to design a reliable nomogram which can aid clinicians in interpreting the results of combined CEA and glucose testing in PCF.

In conclusion, combined CEA and glucose testing in PCF reached high sensitivity and specificity and may thus be considered for implementation into standard clinical practice. A lower cut-off value of CEA increased diagnostic sensitivity. Glucose testing showed high sensitivity and can therefore be used in clinical practice to confirm mucinous PCN.

## Supplementary Information

Below is the link to the electronic supplementary material.

Supplementary file1 (DOCX 14 kb)

## Data Availability

The data that support the findings of this study are available from the corresponding author upon reasonable request. Individual patient data will be shared after de-identification and approval by the study team. Furthermore, a data transfer agreement has to be set up prior to data sharing.

## Abbreviations

AUC: Area under the curve
CEA: Carcinoembryonic antigen
CI: Confidence interval
EUS: Endoscopic ultrasound
FNA: Fine-needle aspiration
IPMN: Intraductal papillary mucinous neoplasm
IQR: Interquartile range
MCN: Mucinous cystic neoplasm
N: Number
NPV: Negative predictive value
PCF: Pancreatic cyst fluid
PCN: Pancreatic cystic neoplasm
PDAC: Pancreatic ductal adenocarcinoma
pNET: Pancreatic neuroendocrine tumor
PPV: Positive predictive value
ROC: Receiver-operating curve
SCN: Serous cystadenoma
SPN: Solid papillary neoplasm
STARD: Standards for Reporting Diagnostic accuracy studies
