# Supplementary material for: Validation of combined carcinoembryonic antigen and glucose testing in pancreatic cyst fluid to differentiate mucinous from non-mucinous cysts
Source: Surg Endosc. 2023 Jan 19;37(5):3739–46. doi: 10.1007/s00464-022-09822-6 (PMC10156886; doi:10.1007/s00464-022-09822-6)
Supplement: Supplementary file 1 — Supplementary file1 (DOCX 14 kb) [file 464_2022_9822_MOESM1_ESM.docx]

| Supplementary Table S1. Accuracy of CEA and glucose to differentiate mucinous from non-mucinous PCN in surgical cohort | | | | | | | | |
| --- | --- | --- | --- | --- | --- | --- | --- | --- |
|  | AUC | 95%CI | *P* value | Cut-off value | Sensitivity (%) | Specificity (%) | PPV  (%) | NPV  (%) |
| **CEA** | 0.82 | 0.66 – 0.98 | 0.001 | ≥ 192 ng/mL  ≥ 20 ng/mL | 56  83 | 100  75 | 100  90 | 44  60 |
| **Glucose**  Glucometer  Laboratory | 0.80  0.74 | 0.60 – 0.98  0.51 – 0.98 | 0.007  0.033 | ≤ 50 mg/dL  ≤ 50 mg/dL | 100  100 | 80  83 | 95  95 | 100  100 |
| **CEA or glucose**  CEA or glucometer  CEA or laboratory glucose | N/A  N/A | N/A  N/A | N/A  N/A | ≥ 192 ng/mL or ≤ 50 mg/dL  ≥ 20 ng/mL or ≤ 50 mg/dL  ≥ 192 ng/mL or ≤ 50 mg/dL  ≥ 20 ng/mL or ≤ 50 mg/dL | 96  96  91  95 | 87  62  87  62 | 96  88  95  87 | 87  83  78  83 |
| **CEA and glucose**  Glucometer  Laboratory | 0.85  0.76 | 0.67 – 1.00  0.53 – 0.98 | 0.002  0.022 | ≥ 192 ng/mL and ≤ 50 mg/dL  ≥ 20 ng/mL and ≤ 50 mg/dL  ≥ 192 ng/mL and ≤ 50 mg/dL  ≥ 20 ng/mL and ≤ 50 mg/dL | 55  85  56  83 | 100  100  100  83 | 100  100  100  94 | 36  62  43  62 |
| Abbreviations: AUC = area under the curve. CEA = carcinoembryonic antigen. CI = confidence interval. dL = demi-liter. mg = milligram. mL = milliliter. N/A = not applicable. Ng = nanogram. NPV = negative predictive value. PCN = pancreatic cystic neoplasm. PPV = positive predictive value. | | | | | | | | |
